# Researches into the Physical History of Mankind

**Published:** 1847-07

**Authors:** 


### Art. IV.

1. *Researches into the Physical History of Mankind.* By James Cowles Prichard, m.d., f.r.s., m.r.i.a., Corresponding Member of the National Institute of France, &c. &c. Third edition.—*London,* 1836-47. Five volumes, 8vo, pp. 2547. With numerous Plates.
2. *The Natural History of Man; comprising Inquiries into the Modifying Influence of Physical and Moral Agencies on the different Tribes of the Human Family.* By James Cowles Prichard, m.d., f.r.s., m.r.i.a., Corresponding Member of the National Institute of France, &c. &c.—*London,* 1843. 8vo, pp. 556. With forty Plates and ninety Wood-Engravings.
3. *Appendix to the First Edition of the Natural History of Man.*—*London,* 1845. 8vo, pp. 40. With six coloured Plates.

If a person, whose knowledge of Mankind is restricted to the individuals of his own nation, and who is consequently unaware of the diversities in complexion, conformation, and habits, which exist among the several races of men, could suddenly be made a spectator of the various conditions which they assume in different parts of the earth, we cannot doubt that his curiosity would be strongly excited, as to the degree of relationship to himself which these races possess.

"If such a person," as Dr. Prichard has well remarked, "after surveying some brilliant ceremony or court-pageant in one of the splendid cities of Europe, were suddenly carried into a hamlet in Negroland, at the hour when the sable tribes recreate themselves with dancing and barbarous music;—or if he were transported into the saline plains, over which bald and tawny Mongolians roam, differing but little in hue from the yellow soil of their steppes, brightened by the saffron flowers of the iris or the tulip;—or if he were placed near the solitary dens of the Bushmen, where the lean and hungry savage crouches in silence, like a beast of prey, watching with fixed eyes the birds which enter his pit-fall, or the insects and reptiles which chance may bring within his grasp;—or if he were carried into the midst of an Australian forest, where the squalid companions of kangaroos may be seen crawling in procession, in imitation of quadrupeds;—would the spectator of such phenomena imagine the different groups which he had surveyed to be the offspring of one family? and if he were led to adopt that opinion, how would he attempt to account for the striking diversities in their aspect and manner of existence?"

This question is capable of being considered under a great variety of aspects. There are many very excellent persons, who think it quite sufficiently answered by the authority of the scriptural narrative; and if we make up our minds to receive with implicit confidence the declarations of the Bible in regard to the common origin of all the races of mankind, the question might *seem* to be decided without the necessity of entering upon any further inquiry. But it is not so in reality. There is a large class of persons at the present time, especially on the other side of the Atlantic, who profess the highest veneration for the Scriptures, and who would look with horror at any attempts to impugn their authority on any subjects regarding which their own prejudices and supposed interests lie in conformity with their teachings: and yet these very persons, to serve their own purposes, get rid of the historical testimony and the various declarations contained in the Bible, in favour of the Unity of Origin of the Human Races,—after the following fashion. They maintain that the *Adamic* race does not include the uncivilized inhabitants of remote regions; and that Negroes, for example, Hottentots, Esquimaux, and Australians, are not in fact *men* in the full sense of the term, or beings endowed with mental faculties similar to our own. By writers of this school it is contended that these and other barbarous tribes are inferior in their original endowments to the proper human family which supplied Europe and Asia with inhabitants; and that, being organically different, they are separated by an "impassable barrier" from the race which displays in the highest degree all the attributes of humanity, and can never be raised to an equality with it. They maintain that the ultimate lot of the ruder tribes is a state of perpetual servitude; and that if, in some instances, they should continue to repel the attempts of the civilized nations to subdue them, they will at length be rooted out and exterminated from every country on the shores of which Europeans shall have set their feet. If the distinct origin of these tribes be admitted,—if we are to regard the Negro and the Australian, not as our fellow-men, brethren of the same great family with ourselves, but as beings of an inferior order,—and if duties towards them were not contemplated, as we may in that case presume them not to have been, in any of the positive commands on which the morality of the christian world is founded, our relations to those tribes will appear not to be very different from those which we might consider ourselves to hold towards the higher races of brutes. We can scarcely imagine a Grotius, or a Puffendorf, or any other great jurist, attempting to determine the *jus belli* or *pacis* between ourselves and a tribe of Orangs, who had just sense enough to pass for men and began to be suspected of the cheat;—which is nearly the true character of the Negroes, if those are right who maintain the doctrines to which we have alluded. And we may go a step farther and assert, that there is in such a case no moral principle which should prevent a hungry wanderer in Negroland or Australia from satisfying his appetite by killing and eating the first native he might happen to meet.

Thus, then, we see that the widest extremes of opinion, and the greatest diversities in those rules of conduct which are founded upon those opinions, may exist among those who profess the most implicit reverence for the Scriptural *dictum*, that "God hath made of one blood all nations of men, for to dwell on all the face of the earth;" for whilst some include under the term *men* all the individuals grouped together by the naturalist under the genus Homo, others assert that this genus includes several speeies, which form a gradation between the highest and most cultivated races of mankind, and those degraded tribes which seem to have more in common with the brutes; the former alone being really entitled to the appellation *Men*, whilst the latter should be designated by some other name indicative of their close affinity to Chimpanzees and Orangs.

But the question may be discussed without any reference to the supposed authority of the Scriptures on this head; and this, not merely by those who reject revelation altogether, but by those who consider the Bible as imparting truths of its own relating to the *moral* not the *physical* history of mankind, for which they entertain the highest veneration; and as not intended to enlighten us upon the present question, any more than upon astronomy or geology. The origin of mankind from a single stock, or from a diversity of stocks, thus becomes a matter of purely scientific investigation; and as such we shall treat it,—not with an entire indifference as to its issue, for we consider it a matter of no trifling importance to arrive at a correct result,—but, we venture to affirm, with an earnest desire to arrive at the *truth,* wherever this may be to be found.

The question may be thus stated. In the *first* place, all the existing races of mankind may be supposed to be the offspring of *one pair;* and it becomes necessary then to account for the diversities which they now present, in physical conformation, in language, and in mental character and social condition. *Secondly,* the existing races may be supposed to have descended from *several distinct pairs,* which originally presented differences amongst themselves, nearly the same with those which now exist amongst the races that seem most remotely related to each other. The first of these suppositions requires that evidence should be given of a very considerable amount of *variability* from the original type—whatever that may have been—amongst the descendants from the common ancestry. The second is based upon the idea that the leading characters which now separate the different races are *permanent,* and must have been presented by their original progenitors. A *third* supposition is perhaps admissible; namely, that the existing races did not all proceed from one stock, but from several; but that these, although scattered over the globe, differed only in the adaptation of certain of their physical characters to the different circumstances of their several abodes, being all possessed of the same essential nature, both mental and corporeal, and all having a certain capacity for variation, so that the prolonged influence of climate, civilization, &c., might in a great degree obliterate the original differences. The moral relations between the several races would, on this last supposition, be as close as on the first, although the same physical affinity or blood-relationship would not exist. We cannot but agree with a recent reviewer of Dr. Prichard's work, in the remark that "the moral rights of men depend on their moral nature; while Africans have the hearts and consciences of human beings, it could never be right to treat them as domestic cattle or as wild fowl, if it were ever so abundantly demonstrated that their race was but an improved species of ape, and ours a degenerate kind of god."* It is in adopting the second supposition that those find their ground of defence, who assert that they are justified in treating as brute beasts certain races which have the misfortune to be dark in complexion and not to come up to their standard of beauty in physical conformation. If the characters of each race *are* permanent, and their respective boundaries fixed by an impassable barrier, the more elevated have a show of justice in claiming for themselves alone the designation of men, and in asserting their right to keep in subjection those degraded tribes which have no sufficient title to the name.

The question of the *permanence* or the *variability* of the races of men is therefore one of fundamental importance; since, if the former be established, not only is a strong argument afforded for original diversity of parentage, but (what is of far more importance in our apprehension) we are almost forbidden to hope that the races which we at present regard as inferior, can be in any way raised to our own level; whilst if the latter

* New Quarterly Review, No. xv, p. 131.

can be proved to be the case to a sufficient extent, the greatest difficulty in the way of the idea of the community of origin of mankind is at once removed, and we are encouraged in the belief that there is no race, however degraded at present, that may not be ultimately raised to the highest elevation of which humanity is capable. In the discussion of this question, a vast number of different topics are involved. The number of cases in which positive historical testimony can be brought to bear upon the question of the identity of origin of two or more races at present exhibiting characters of a very diverse nature, are comparatively few; and appeal is necessary to Philology, a branch of science with which Physiology would seem to have no direct relation, in order to obtain the requisite information. The inquiry into the nature and affinities of the several languages at present or in past times spoken in different parts of the globe, has been carried on of late years with great earnestness, especially by the *literati* of Germany; and notwithstanding discordances on minor points, the leading philologists of the day are fully agreed upon certain general principles, which we think it well here to quote, for the sake of exhibiting to our readers the *nature* of one of the sources of information from which Dr. Prichard draws a large proportion of the materials of his argument.

"On examining the relations of language which are said to display marks of resemblance or conformity, two very different series of phenomena are discovered, which lead to very different results. Languages of neighbouring nations, or of nations long and intimately connected by local proximity, by traffic and commercial intercourse, or by political bonds, exhibit marks of such connexion in their vocabulary, or in the possession of a great many words in common. Of this description is the extensive resemblance in words between the French and English languages.* In the languages of nations who may have come into a similar nearness of intercourse while in different degrees of social culture, when the one people possessed many arts and the knowledge of very many objects, of which the other were wholly destitute, it is evident that a much more extensive resemblance would take place than that which is discovered between the French and English. But this species of resemblance or partial identity in the vocabulary could never approach to what is termed a family relation of languages; that is, such a kind of connexion between them as affords proof of a common origin in the nations to which they belong, as in the instance of the English language compared with the German. The first and most important feature indicative of family relation between languages, is analogy in grammatical structure, and in the laws of combination, or, as we may so term it, the mechanism of speech.—Languages supposed to have been originally cognate have, in some instances, lost every other token of relationship except this. It generally happens, however, that grammatical affinity between languages is accompanied by a near resemblance in a certain part of the vocabulary. Occasionally this extends only to a comparatively small number of words; but they are words of a particular class, namely, such as serve to represent the ideas of a people in the most simple state of existence. Such are the terms expressive of family relations,—father, mother, brother, sister, daughter; names for the most striking objects of the visible universe; terms distinguishing different parts of the body, as head, feet, eyes, hands; nouns of number, up to 5, 10, or 20; verbs descriptive of the most common sensations and bodily acts, such as seeing, hearing, eating, drinking, sleeping. As no nation was ever found destitute of similar expressions, and as we know by the observation of facts even more than by the probability of the case, that tribes, however

* I have selected this example as the most familiar instance. It is liable to the objection that the French and English do not belong to originally distinct families of languages. The Anglo-Saxon and the Norman French were, however, so different, that in a practical point of view this instance answers my purpose as well as any other.

rude, do not exchange their own stock of primitive words for those of a foreign idiom, it may be inferred that dialects which correspond in these parts of their vocabulary were originally one speech, or the language of one people." (Natural History of Man, p. 182.)

We have in the case of the languages of the several races of which the great mass of the population of Europe is composed, an example of both these kinds of conformity; namely, in grammatical construction, and in what have been termed primary words. And this conformity not only links them to each other, but connects them with the more ancient languages of central Asia, the Sanscrit or original language of the Hindoos, and the Zend or earliest idiom of the Medes, Persians, and Bactrians; and these two are so intimately related to each other as to leave no room for doubt as to their common origin. On the other hand, the languages of America are entirely different from those of the Old World in both particulars. Their grammatical structure is distinguished by a very striking feature; namely, the peculiar manner of forming compound words, which has been called "agglutination." This is very different from the ordinary composition of words in the languages with which we are most familiar; the process being to make up new compounds from a number of *small fragments* of simple words, and then to treat these compounds as if they were simple vocables, mutilating or contracting them to form other aggregate words. Thus when a Delaware woman is caressing or playing with a little dog or cat, or some other young animal, she will often say to it *kuligatschis*, meaning, "give me your pretty little paw." The word is thus compounded:—*k* is the inseparable pronoun of the second person, representing *ki*, and meaning either "thou" or "thy;"—*uli* is part of the word *wulit*, meaning "handsome" or "pretty;" *gat* is a part of the word *wichgat*, meaning "leg" or "paw";—and *schis* is a diminutive termination. This peculiarity, especially consisting in the introduction of very small portions of words into the compound term, and in the large capacity of the latter, distinguishes them from all known languages of the Old Continent, except perhaps the Euskarian; and even in this the agglutinating process is rarely carried to so great an extent as it habitually is in the American idioms. "The only Greek compound words," says Dr. Prichard, "which are comparable with those of the Lenape, are the long fantastical epithets used in burlesque by Aristophanes in some of his comedies. If we could imagine the main stock of words belonging to a language to be made up of such materials, and adapted not to poetry either serious or ludicrous, but to the purposes of ordinary conversation, we should perhaps have an idea of the nature of the words which furnish names for objects in the American idioms. The following is another striking feature in the physiognomy, so to speak, of the American language.

"These languages, like the idioms of perhaps all nations in a similar state of society, may be said to be rather more subjective than objective. The prevailing impulse in a rude people of hunters and warriors is, not to discriminate the qualities of external objects, but to give vent to the internal feelings, passions, and desires of their own minds; their egoism, or personal volition and action, is ever uppermost, and the predominant movement of their mental life. Circumstances and externals are secondary, and draw but little of their attention. Hence verbs or words expressive of emotion, will, and action, are the principal words in these languages, and are developed in the greatest variety of forms. There is a constant tendency to involve in the expression of the verb, and to denote in one word as much as possible, the circumstances and conditions of the agent, and the external relations of the act he has performed, or is perhaps about to perform. The alterations in the forms of the verbs thus induced go further than the changes of mood, tense, and conjugation, which are common in different degrees to most languages. They are contrived to express, without the use of distinct words, as many varieties and shades of meaning as possible. Even physical objects, especially where pronouns are used, are pointed out without being expressed, through some slight alteration in the forms of verbs." (Physical History of Mankind, vol. v, p. 312.)

Some very extraordinary compounds are thus elaborated to express abstract ideas, which in other languages are designated by simple terms. Thus *wulamœwagan,* which means "the truth," is derived from the verb *wulamœ,* "he has spoken the truth" and the formative *wagan,* which resembles the English *ness;* and *wulamœ* itself is compounded of three words, a pronoun, a portion of the term meaning "good words," and another which implies the act of speaking. By the insertion of a portion of the verb *glistam,* which means to "hear" or "listen," we get the compound *wulistamœwagan,* which means belief in what is said by another.—These examples will suffice, we think, to convey to our readers some idea of the peculiar character of the American languages, so far as regards their construction. This feature serves not merely to distinguish them from the languages of the Old World, to such a degree as to show that the two families of nations must have been very early separated; but it also serves to connect the different American tribes, from Greenland to Cape Horn, by a common bond of relationship, in spite of a very considerable diversity in their primary words and in the roots of their vocabulary. This diversity, —extending in some cases, it has been thought, to total dissimilarity,— might appear to forbid that idea of a common origin which has been founded on similarity of grammatical structure. But as Dr. Prichard has well pointed out, there are peculiarities in the very nature of the American languages, which are likely to produce great varieties in words, and to obliterate in a comparatively short period the traces of verbal resemblance. We quote his remarks on this subject nearly in full, because we wish to point out to our readers the vast number of the considerations that enter into the inquiry on which we are engaged; and because we shall not again revert to the philological principles which are especially involved in it.

"1. The great length of words in the American languages is unfavorable to the preservation of resemblance between vocabularies of separated tribes...... It is impossible that the memory can be so tenacious of long polysyllabic words, as of shorter ones; and if the habit prevails of truncating words at their beginnings and endings without rule or limit, it is very obvious that any resemblance that existed between them originally may soon be lost. In the languages of Asia and Europe, roots are either monosyllables, or at most dissyllables; and these are not so much lengthened out by additional syllables as the American languages. Hence they are better retained in memory. It is in fact principally in words of not more than two syllables, that those resemblances exist, which are so striking when the different members of the same family, as of the Indo-European or the Syro-Arabian group of languages, are compared.

"2. It is easy to perceive that the agglutinative system of formation, which is the principle of structure belonging to the American languages, must tend effectually to destroy resemblance. We have seen that it is the custom in conversing in the Delaware language to aggregate some parts merely of as many words as may occur to the mind of the speaker, in order to construct for temporary use a compound word that shall comprehend all the circumstances of any action. ......It must soon happen among people who have the habit of thus expressing themselves, that only those persons who live near to each other and have some acquaintance and frequent intercourse, can readily make themselves mutually intelligible. They must apparently know something of each other's thoughts and habits of mind before they can understand such casual and arbitrary enunciations of meaning, in which words newly coined for the occasion are of perpetual occurrence........

"3. Another cause which has been observed to render obscure and difficult of detection, the original connexions between American languages, and which must always have a tendency to increase or create differences in the vocabulary of idioms really cognate in their origin, is the imaginative and rhetorical disposition of the native people of the New World. In a barbarous state of society, and principally in one of early and imperfect but growing refinement of mind, the imagination has more influence in the formation of language than in a more advanced stage. When scientific accuracy and precision of thought and expression are required, and it becomes the habit of men to aim at such acquirements, the exercise of the imagination is restrained within very narrow limits. Observation and discrimination are employed. But figures and metaphors abound in the discourse of simple people; and hence the eloquence for which the American nations are celebrated. As a matter of fact it is the habit of the American tribes to substitute for words epithets which become conventionally established as ordinary terms. Dr. Scouler has observed that in the languages of the north-west coast in particular, the names even of simple and familiar objects, such as sun, moon, day, night, &c., are not simple nouns or names, but not unfrequently compound words or epithets. In this case he suggests, unless we possess an intimate knowledge of the influences of the verbs, and the nature of the indeclinable particles, we might mistake two idioms nearly allied for primarily separate languages." (Physical History, vol. v, p. 320.)

Thus we are informed by Dr. Scouler that among the tribes of the north-west coast, the names given to articles introduced among them by European traders, are not, as is common among rude nations, derived from the foreign designations of the same objects; but they are descriptive epithets, formed in their own languages, and consequently different in the idiom of each particular tribe. Thus, the name of a gimlet among the Chemesyans is a compound formed from the noun which means "a hole" or "aperture," and a verb signifying "to make." The compound name means "a hole-maker."

It is evident throughout his work, that Dr. Prichard sets a primary value upon fundamental affinities of language as indicative of family relationship amongst groups of nations; and that he assigns to it a vast superiority over the indications afforded by physical characters. The impossibility of drawing any correct inferences from the latter alone, will become apparent, we think, when our readers shall have followed us through our review of Dr. Prichard's investigations in regard to their variability. But we have looked in vain through his work for any corresponding general examination of the kind of evidence derivable from language; and his readers are obliged to take for granted the philological principles on which he has conducted the details of his inquiry. Now as very few of them are sufficiently versed in the science of glottology, to be able to judge for themselves with regard to the value of these principles, without some further elucidation of them than has been afforded in either of the works before us, we feel that the cogency of Dr. Prichard's arguments is considerably diminished by the numerous exceptional cases, in which from conquest, migration, and other causes, an *entire* change in the language of a nation has taken place, without any considerable intermixture with another race. Thus to take a very familiar case, that of the Jews;—their dispersion through almost every country in the world has caused each individual to learn and to speak familiarly the language of the nation in the midst of which he has been brought up; yet their national descent has been preserved more purely than that of any race not isolated by local separation. The preservation of their original language is an accidental circumstance, arising out of their religious peculiarities; and it is easy to foresee that, if the Jewish nation were converted to Christianity, the Hebrew tongue might soon be reckoned among the languages now extinct and only known to the learned. If we were to rely solely upon the existing languages of Britain, France, and Spain, and had no guidance from history with regard to the mode in which they have been formed, we could scarcely avoid falling into errors of the greatest importance as to the original stocks of the nations at present inhabiting those countries. Consequently, in cases where we are totally destitute of any trustworthy history, and have either vague tradition alone to direct us, or have no historical data whatever, it appears to us that great caution must be exercised in drawing inferences from analogies or diversities in the languages of different nations with respect to their degree of family relationship. Doubtless there are collateral circumstances, which may afford us great assistance in the inquiry; and which may give to inferences founded upon fundamental community of language, between certain groups of nations, a degree of weight which they do not elsewhere possess. And we believe that we shall be able to show that this is especially the case in some of the instances in which there is a wide diversity in physical characters; so that, when the variability of the latter within certain limits is admitted,—as we conceive it *must* be, if the question be candidly and philosophically investigated,—the *general* superiority of the glottological over the physical test must be freely conceded, even although the results of the application of the former may be doubtful in particular cases. The relative character of the two may, as it appears to us, be stated somewhat as follows.

We shall suppose a family or group of nations to spread from a common centre; carrying with them the language, habits of thought and expression, traditions, religious observances, &c., derived from their common parentage; and dispersing themselves through countries that differ considerably in climate and in those other conditions which affect the social existence of the human race. Now all experience shows that amongst nations thus separated considerable diversities soon present themselves, and that language begins to undergo modification sooner than anything else, unless it have been previously fixed by a national literature. Dialects soon spring up, distinguished by a different method of pronouncing the same words, which subsequently gives rise to a fixed diversity in the mode of spelling them; and new words are created to express new requirements, which words will obviously be peculiar to the nation or tribe amongst which they have originated. As some of these diverging tribes, however, come into contact with others which have spread from a different centre, and intercourse is established between them, their language receives an admixture of elements altogether foreign; and thus a still greater diversity is created amongst the several dialects. By a long continuance of such changes this diversity may become so great, as to cause the languages of nations which have diverged from a common centre and have really had the same ancestral origin, to appear to a superficial observer to have nothing in common. But the experienced philologist will look for similarity of *roots* amidst diversity of *inflexions;* for correspondence in "primary words," amidst dissimilarity of terms expressive of less necessary ideas; and above all for a fundamental accordance in the grammatical construction or plan of mechanism of the language, in the midst of great variety in the mode in which the details of the plan are worked out. Where no greater change has been effected in any group of nations, than that which results from contact and intercourse with others, we believe that clear evidence of their original relationship may be derived from these points of accordance. The evidence is of course less clear in proportion to the deficiency of *verbal* coincidence; but it seems to lose but little of its force, where, as in the case of the American languages, the peculiarity of grammatical construction is marked with extraordinary force, and where (as is pointed out in the quotation which we have made from Dr. Prichard's remarks upon them) the very nature of this construction and of the genius of the language is such as to produce a speedy verbal discordance.

On the other hand, although the physical characters of the several nations thus diverging from a common centre, may not for centuries present any decided change, we think that we shall be able to draw from Dr. Prichard's researches, and from other sources, clear evidence that the lapse of centuries *may* alter them in a degree so considerable, as altogether to break down any distinction that may have once existed between their common original and the original of some other group of nations. Of course, the greater the change of climate and other external conditions encountered by particular tribes during their divergence from each other, the greater would be the modifications we should be entitled to expect, if these modifications are really induced by external causes: and such, we think, will prove to be the case; some nations presenting, as nearly as can be ascertained, the physical characters of the original stock; whilst others have widely departed from it. Hence, if the tendency of such variations be to bring the different races of men that cover the globe into conformity with its varying physical conditions, we should expect to find that the bodily characters of the different races have a much closer relation to the circumstances in which they now live, than they have to the characters of their supposed parents. There is an ample accumulation of evidence to this effect; with regard at least to some of those characters on which the greatest stress has been laid as means of determining the affinities of races,—namely, the colour of the skin, and the form of the cranium.

We have here taken for granted, however, the results of those physiological inquiries to which a considerable portion of Dr. Prichard's treatises is devoted; but as we are particularly desirous that our readers should be well informed upon this department of the subject, we shall now proceed to examine into the evidence which has led us to assert with so much confidence, that the distinctive characters of the human races are *not* fixed and invariable, but are liable to undergo modification through the agency of various external conditions, as well as to become altered from time to time by variations, which, in the absence of any ostensible modifying influence, are considered as *spontaneous.*

In following out any inquiry of this nature, it is desirable to commence with a comprehensive survey of the phenomena which have relation to it amongst other tribes of animals. We do not say that it *might* not be satisfactorily pursued with reference to the human races alone; but there will be more probability of a decisive result, when that result is not capable of being endangered by arguments derived from collateral sources; and we consider the analogical investigation into which Dr. Prichard has entered, with such completeness as nearly to exhaust the subject, as one of the most valuable portions of his treatise, although it might at first sight appear too digressive from its main purpose. We have more than once had occasion to point out, that amongst the different species of plants and animals, there is a very wide diversity in regard to their respective *capacities for variation;* the several individuals among some being all very closely conformable to one type; whilst in others, individuals that are known to have the same parentage exhibit differences in external characters, and even in internal structure, which would have been regarded, if they had occurred amongst races known to have less capacity for variation, as unequivocally indicative of diversity of origin. In the species which have least capacity for variation, we find (as a necessary consequence) the *least* adaptiveness to external conditions; they are limited, therefore, in their distribution to the parts of the globe which supply those conditions, any decided change of these being fatal to them. On the other hand, in those which have *most* capacity for variation, that capacity manifests itself in the peculiar adaptation which their physical constitution undergoes to circumstances as they change; and also in the spontaneous origination of peculiarities that cannot be traced to the influence of those circumstances. Thus, when animals that are naturally inhabitants of cold or temperate climates, and have a thick protective covering of wool, exchange this for a thin covering of crisp hair, when they have been transported to a tropical region, we at once see that the variation is influenced by the external change of temperature. On the other hand, when a new race arises with a number of fingers, toes, or vertebræ, different from that of its parent, we must set down the occurrence as another manifestation of the same capacity for variation, only that we cannot here trace its connexion with any external influences. It will generally, if not always be found, that the races which show the greatest disposition to be affected by external changes, are precisely those that show most tendency to that divergence from a common type, which must (for the present at least) be regarded as spontaneous. Such is the case, for example, with our domesticated animals; which, like plants improved by cultivation, derive their capability in usefulness to man, in part from the degree in which they may be modified by external influences, and in part from their disposition to pass into spontaneous variations, of which those that seem likely to become useful to him are speedily perpetuated by his interference.

Of the greater number of our domesticated races, the origin is lost in antiquity so remote, as to prevent the possibility of our obtaining direct historical testimony as to their ancestry. There are few cases in which we can point to any existing *really wild* race, that can with any certainty be considered as the representative of the parental type, still uninfluenced by domestication. We know not what has become of such;—whether they have been entirely subjugated by man, so that we at present only know the species in their altered forms, or whether the wild representatives of the original species are really so different from their reclaimed descendants, as to be unhesitatingly separated from them by the naturalist, although really possessing a common ancestry. Thus it is still an undecided question, whether the primeval dog was anything else than a wolf; the wolf being the original wild form, and the various breeds of dogs the more or less modified types of the same species. There are indeed *wild* oxen, sheep, goats, and horses; but most, if not all of these, are tribes which appear to have returned in some degree to their original state after having been more or less completely domesticated; and we are ignorant of the time and circumstances under which most of these races became wild, and of the particular breeds from which they have descended.

Still the phenomena which have taken place within the historic period, and of which there is indisputable proof, afford ample evidence of the wide range of variation, through which certain species of animals are capable of passing, in the course of even a few generations; and show that although the tendency to spontaneous variation may seem to have nearly exausted itself heretofore, in the production of the most divergent forms, there still remains enough to originate new races, distinguished by well-marked peculiarities of conformation, even under our own eyes. Some of our most valuable information upon this subject is derived from the changes which have taken place in the races of domesticated animals, introduced into the West Indies and South America, by the Spaniards, three centuries since. Many of these races have multiplied extremely on a soil and in a climate congenial to their nature; and several of them have run wild in the vast forests of America, and have lost all the most obvious appearances of domestication. The wild tribes are found to differ physically from the domesticated breeds, from which they are known to have originated; and there is good reason to regard this change as a partial restoration of the primitive characteristics of the wild stocks, from which the tame animals originally descended. The comparison of these wild races with our domesticated breeds affords much information which is important to our present argument; and we shall now proceed to give a summary of the facts, which are drawn by Dr. Prichard from the valuable memoir of M. Roulin, whose researches relate to New Grenada and Venezuela, and from the well-known and justly-esteemed work of Don Felix de Azara on the Natural History of Paraguay.

We commence with the hog; which was introduced into St. Domingo by Columbus on his first discovery of that island in 1493, and which was speedily carried into the continent of South America; multiplying with extraordinary rapidity in both situations.

"These animals, wandering at large in the vast forests of the New World, and feeding on wild fruits, have resumed the manner of existence which belonged to the original stock; and their appearance nearly resembles that of the wild boar. Their ears have become erect; their heads are larger, and the foreheads vaulted at the upper part; their colour has lost the variety found in the domestic breeds, the wild hogs of the American forests being uniformly black. The hog which inhabits the high mountains of Paramos, bears a striking resemblance to the wild boar of France. His skin is covered with thick fur, often somewhat crisp, beneath which is found in some individuals a species of wool. From excessive cold, and defect of nourishment, the hog of that region is of small and stunted figure. In some warm parts of America, the swine are not uniformly black, as above described; but red, like the young pecari. At Melgara and other places, there are some which are not entirely black, but have a white band under the belly reaching up to the back; they are termed *cinchados*." (Natural History of Man, p. 30.)

Now these few facts might alone serve as the groundwork of a very important argument. It was long ago pointed out by Blumenbach, that the difference between the cranium of the wild boar and that of our ordinary swine, is at least equal to that which exists between the cranium of the Negro and the European; yet we see that the change from one form to the other may take place in the course of a few generations. The difference between the thin and sparse bristles of the ordinary swine, and the thick fur with subjacent wool, with which the hogs of the mountains of Paramos are covered, is at least equal to that which exists between these tegumentary appendages in any races of men. And the restoration of an uniform black colour throughout the inhabitants of extensive districts, is an equally significant proof of the influence of external circumstances in modifying this character. It has also been pointed out by Blumenbach, that the varieties of swine in other countries, all referable to a common stock, exceed in divergence the widest departures from any one type of human conformation. Thus swine with solid hoofs were known to the ancients, and large breeds of them are found in Hungary and Sweden, as also in some parts of England. In like manner, the European swine, first carried by the Spaniards in 1509 to the island of Cubagua, have degenerated into a monstrous race, the toes of which are half a span in length; and in some other breeds the hoof is divided into five portions.

Horned cattle were introduced into St. Domingo in the second voyage of Columbus; and thence into the Spanish settlements on the mainland, whence they have spread over the South American continent. The herds of wild cattle, which have descended from these, are distinguished, like the hogs, by their uniformity of colour; the upper parts being of a brown red, and the rest of the body black. That climate alone does not produce this effect, is evident from the fact that the herds of domesticated cattle in the same country exhibit the usual variety of hue.

"In some of the hot provinces of South America, M. Roulin informs us that a variety of ox has been noted for an extremely rare and fine fur. These oxen are termed 'pelones.' The variety is reproduced or descends in the stock; but is not cultivated, because the pelones are too delicate in constitution to bear the cold of the Cordillera, to which the cattle are driven for the provision of the towns there situated. The pelones evidently constitute a variety adapted to a particular climate. Oxen of other breeds often perish when driven into the same provinces, and are with difficulty assimilated to the climate, or *acclimatised*. In the same hot countries, a variety is sometimes produced with an entirely naked skin, like that of the dogs without hair found at Calongo on the coast of Guinea. These cattle are called Calongos; they are very delicate and weak. This variety never makes its appearance in cold districts.—Another remarkable fact relative to the oxen of South America is recorded by M. Roulin. In Columbia, the practice of milking cows was laid aside owing to the great extent of farms and other circumstances. In a few generations, the natural structure of the parts, and withal the natural state of the function, has been restored. The secretion of milk in the cows of this country is only an occasional phenomenon, and contemporary with the actual presence of the calf. If the calf dies, the milk ceases to flow; and it is only by keeping him with his dam by day, that an opportunity of obtaining milk from the cow by night can be found. This testimony is important, by the proof it affords, that the permanent production of milk in the European breeds of cows is a modified function in the animal economy, produced by an artificial habit continued through several generations." (Natural History of Man, p. 34.)

In the year 1770, as we learn from Azara, a hornless bull was produced in Paraguay, which has been the progenitor of a race of hornless cattle that has since multiplied extensively in that country. We need not refer to the remarkable differences in the form, size, and direction of the horns which exist amongst the domesticated breeds of our own country; since with these our reader must be familiar. But we may add to the foregoing statements, a notice of the Zebu or Brahmin ox, which inhabits the East Indies, and is found also in Madagascar and the eastern coast of Africa. It is chiefly remarkable for its large fatty hump; the size of which, however, varies with the degree of repose and the amount of food which the animal enjoys. This animal is distinguished by a docility fully equal to that of the European ox; and it is thought by many naturalists to be of the same species. Whether or not this be the case, it has the same tendency to pass into varieties; for numerous races are to be met with, differing in size from that of our largest cattle to that of a young calf, and having variations also in form and aspect. Further, it now seems to be the general opinion, that our domesticated breeds are the descendants of a wild species, which formerly inhabited the forests of central Europe, and which was described by Cæsar and other ancient authors under the name of *Urus*.* This appears, from historical evidence, as well as from the remains preserved to us, to have been a much more powerful animal than our existing races; being especially distinguished by the size and direction of its horns. In a specimen found at Melksham, the distance between the ends of the bony cores was four feet; and the space between the horns at their greatest distance must have been much more. The horns bent forwards and downwards. The difference between the ancient urus and our domesticated breeds was not greater, however, in this respect at least than that which exists among the different breeds of the half domesticated buffaloes. Thus the arnee of India, which is referred by the most competent authorities to the same stock with the ordinary buffalo, has horns which often measure from four to six feet in length, and ten feet between the tips.

The sheep which were transported into America by the Spaniards, have not multiplied so extensively as the oxen and swine; and in many parts they are scarcely to be met with. The following fact, however, regarding the sheep of one of the valleys of the Cordillera, shows the marked influence of climate in altering their woolly covering.

"Wool grows on the young lambs nearly as in temperate climates; if shorn, it sprouts again, and the fleece is formed as usual. If neglected, however, it forms itself into a large tufted mass, which breaks off in shaggy portions. When it comes off, there is found beneath, not fresh wool, nor a naked and diseased skin, but a short fine hair, shining and smooth, like that of the goat in his best state; and this remains permanent, the wool never reappearing." (Natural History of Man, p. 37.)

* The animal now known under the name of urus or aurochs, is of a different species.

We have ourselves observed the same kind of modification in the covering of the sheep inhabiting our West India colonies; and we believe that we are correct in stating, that a residence of two or three years is sufficient to produce it in sheep transported from temperate climates. So completely similar is the hairy covering of the sheep and the goat in those climates, that the two animals cannot be distinguished in any other way than by their general conformation; and this, too, from the general absence of any considerable accumulation of fat in the bodies of the sheep, is not nearly so distinctive as it is with us. The goat in South America has become more agile, of more slender make, with the head better formed, and bearing smaller horns than in Europe. The most marked sign of domesticity in our European goats, viz. the large size of the teats, has completely disappeared in the goats, as in the cows, of South America. We are informed by Azara, that sheep and goats bear twice in the year in South America, and produce three lambs or kids annually.

The sheep is one of the most anciently domesticated animals, and it is one in which great varieties display themselves. We have in our own country numerous breeds, differing as to their stature, the texture of their wool, the presence or absence of horns, &c. But these are by no means so unlike each other, as are the various breeds of Asia and Africa, which are regarded by naturalists as having a common specific character. Thus we find in Spain a breed of sheep distinguished by the length and straightness of the hair, and by their long spiral horns. In the breeds spread through Persia, Tartary, and China, the tail seems replaced by a double spherical mass of fat, which forms a most awkward excrescence on the rump, and is nearly destitute of hair. These sheep, when transferred to the cold dry pastures of Siberia, lose their accumulations of fat after a few generations. The sheep of Syria and Barbary, on the other hand, have an accumulation of fat in the tail itself, which is long, and sometimes attains a weight of from 70 to 100 lbs.

"Now breeds of sheep are frequently formed in different countries in which particular qualities predominate, according to the preference of the breeders. This is done, partly by crossing or intermixing races already constituted and well known, but in great part also by selecting individuals from the stock, in which the particular qualities are more marked than in the generality of the same breed. In these instances, the natural or congenital variety, which the individual animal displays perhaps for the first time, becomes perpetuated by the hereditary transmission of such characters, which is the law of the animal economy. A striking instance of this fact is to be found in the origination of the new breed of sheep in the state of Massachusetts, which has been noticed by many writers in connexion with this subject. In the year 1791, a ewe on the farm of Seth Wright gave birth to a male lamb, which, without any known cause, had a longer body and shorter legs than the rest of the breed. The joints are said to have been longer, and the fore-legs crooked. The shape of this animal rendering it unable to leap over fences, it was determined to propagate its peculiarities; and the experiment proved successful; a new race of sheep was produced, which, from the form of the body, has been termed the otter breed. It seems to be uniformly the fact, that when both parents are of the otter breed, the lambs that are produced inherit the peculiar form." (Natural History of Man, p. 46.)

The history of the propagation of this breed offers some instructive facts. In the first year that this peculiar race was used for breeding, only two lambs were obtained with the same peculiarities. More were obtained in subsequent years; but it was only when they became capable of breeding with each other, that the new race became permanent, the variety having previously a tendency to merge into the ordinary type. Now in the human race, it is not uncommon to meet with families, distinguished by some peculiar feature, or by a well-marked departure from the ordinary conformation, such as the possession of six fingers on each hand, and six toes on each foot. If such were to intermarry exclusively with one another, there can be no reasonable doubt, that the children would invariably exhibit the same peculiarity; and the six-fingered race, which now tends, whenever it is originated, to merge in the prevailing five-fingered type, would then become permanent. When it is considered that the influence of a scanty population, in the early ages of the world, by isolating different families from each other, and causing intermarriages amongst even very near relatives, would have been precisely the same with that which is now exercised by the breeders of animals, we can understand why the varieties which *then* arose should have had a much greater tendency to become permanent, than most of those which *now* present themselves.

We need not advert to evidence of a parallel kind, furnished by the known variations of several other species of domesticated animals; but we must say a few words in regard to the dog, which, of all known species, seems to be the one most capable of passing into widely-divergent varieties. These varieties differ extremely, not merely in their stature, the form and proportions of their body and limbs, the nature and colour of their hairy covering, &c.; but also in a point which elsewhere seems more unalterable, namely, the conformation of the cranium, the size of the brain, and the development of the organs of the senses. Thus, to advert to a few breeds only, we find in the Dingo of Australia a cranium, differing but little from that of the wolf.

"In both the head is very flat, and the cavity which contains the brain has comparatively very little space. This arises from the flattening of the temporal and parietal bones; which, from their outer and lower margins, pass almost in a level plane towards the median line, where they join the opposite bones with very little elevation, thus forming a flattened roof for the cerebral cavity. The Danish dog, and the mastiff, resemble the Australian in the shape of their heads, and they display as little development of intellect or sagacity. The terrier and the hound differ from the preceding breeds, in having the parietal bones much more arched, and allowing a larger space for the brain. The greyhound has a larger muzzle and smaller frontal sinuses than the hound; and the sense of smell is proportionably deficient in this breed. The shepherd's dog, which displays much greater sagacity than the hunting dogs just mentioned, and which Buffon very erroneously considered as the least modified by domestication, has a very considerable capacity of the cranium. The temporal bone in the head of the shepherd's dog is not flattened from the inferior margin, or rounded off with a trifling degree of arching or elevation towards the opposite side. In the shepherd's dog, the bones rise perpendicularly to one half their vertical extent, and then become arched over the space occupied by the brain. The wolf-dog resembles the shepherd's dog. Again, in the spaniel and water-dog, the capacity of the cranium is much greater than in the shepherd's dog; and these races, in all their varieties, are remarkable for a great development of the frontal sinus, which is so considerable, as to give the outline of the forehead a direction almost perpendicular to that of the nasal bones; the lower jaw is very much bent. The head of the bull-dog differs remarkably from all the preceding varieties; the posterior parts of the system of facial bones are situated higher than the muzzle, and the jaws have a curved direction; the muzzle is shortened, and its breadth greater as four to three. The cranium of the bull-dog is much less capacious than that of the shepherd's dog; and the parietal bones, instead of being arched, bend towards each other almost at right angles. The docility of these races evidently bears a due proportion to the capacity of their skulls. The wolf-dog, and the spaniel and water-dog, display wonderful intelligence, and seem to understand the voice of man." (Natural History of Man, p. 57.)

It has been asserted by some naturalists, that the chief varieties of the dog ought to rank as distinct species; but there are several objections to such a view. And this question is one of peculiar interest to the anthropologist, since the very same mode of inquiry ought to be pursued in both instances; and that which is freely admitted to be the right method of arriving at a decision in the one case, must be the legitimate ground for determining the other. In the first place, then, it is extremely difficult, if not quite impossible, to draw definite lines between the several breeds of dogs; owing to their strong tendency to mutual approximation. The best-marked or typical forms of each kind may be distinct enough; and yet, even without any crossing of breeds, it is found on a close examination that intermediate forms not unfrequently present themselves, possessing the distinctive characters of their race in a modified degree, and even blending them with those of some other. Moreover, if we once begin to form distinct *species* out of the different breeds of dog, we cannot stop short with five or six, but must go on, as Frederic Cuvier showed, to at least fifty. Again, the different races all breed freely with each other; they have the same period of utero-gestation; and, when they escape from the dominion and influence of man, and go back to their wild unreclaimed state, they all return to a common form, lose their variety of colour and marking, and assume new habits adapted to the circumstances in which they live. It is a curious circumstance that they generally lose the power of barking, which appear to be an hereditary accomplishment, first acquired under the influence of man. The proper wild dogs, such as the Dingo of Australia, do not bark; they only howl. The dogs introduced by the Spaniards, which have run wild in South America, have lost the habit of barking. The progeny of the dogs, left by the Spaniards in the island of Juan Fernandez, before Lord Anson's time, were never known to bark. Two dogs brought to England by Mackenzie from the western parts of America, could never bark, and continued to utter their habitual howl; but a whelp, bred from them in England, learned to bark. It is a curious observation of M. Roulin, that the cats also in South America have in like manner lost those *miaulemens incommodes*, which so often disturb the nocturnal repose of the inhabitants of Europe. A distinguishing feature of all the races of dog, varying, however, with their degree of intelligence, is their disposition to attach themselves to man. This, however, seems to return to a dormant condition in all the races that have run wild; but to remain capable of being aroused in the course of a generation or two. For these and other reasons, it appears to be a conclusion from which there is no legitimate means of escape, that all the breeds or races of dog are varieties of one common specific type. The question is, what is that type?

There is no race of wild dogs at present existing, which can be regarded with any certainty as having kept up the original stock, without having been at any time altered by the influence of man. Even the Australian dingo, which seems the least humanised (if we may use the expression), is probably the descendant of a once domesticated race; since there are many considerations which induce the zoologist to believe that it is not properly a native of New Holland, like the marsupials and monotremes which there represent the mammalian type, but that it was introduced there by the agency of man. Now it is a fact that seems common to the Australian dingo, the dhole of India, and other partially wild races of dogs, that their general conformation approaches that of the wolf. "In proportion, as they are more wild, they exhibit the lank and gaunt form, the length of limbs, the long and slender muzzle, and the great comparative strength, which characterize the wolf; and the tail of the Australian dog, which may be considered as the most remote from the state of domestication, assumes the slightly bushy form of that animal." (Bell's British Quadrupeds, p. 197.) The uniform dull-brown hue, moreover, which all the wild races of dogs present, much resembles that of the common wolf. It has been objected that the wolf does not exhibit that character, which is so remarkable in all the races of dogs,—a disposition to attach itself to man. Even the wild breeds of dogs are easily brought under subjection, and are made useful to him in various ways, which could not be the case if they had the same savage disposition as the common wolf. But it has been proved that the wolf is much more capable of domestication than is commonly supposed, if taken young from its wild state and brought under the influence of man; and that it then displays as much attachment to its master, and remembrance of kindness shown it, as any dog could do. So that there is no difficulty in understanding how, by a continuance of this influence through successive generations, the character of the race may become so permanently changed that the traces of former domestication may not be altogether lost, even in breeds which have returned to their wild state for centuries. There is no reason why the *psychical* character of a peculiar aptitude for domestication should not manifest itself as the special feature of a variety, like any *physical* character, such as the remarkable conformation of the otter breed of sheep; or why, to put the matter in a still more definite form, the first manifestation of the variability of the species of wolf, should not be the production of a race disposed to attach itself to man, from which race, under the continued influence of domestication, an almost infinite variety of new breeds has arisen, differing in the number of vertebræ in their tails, in that of their toes, and even in that of their molar teeth. These last departures from the ordinary type exist, not merely in particular races most remote from it, but also within the limits of single varieties or breeds, just like the occasional appearance of six-fingered races among various nations of men. They cannot, therefore, be admitted as specific differences; and they serve to prove how *very* wide are the limits of variation in this species.

The question of the specific identity of the dog and the wolf is at present undecided; and it is generally admitted that the determination must be founded chiefly upon the characters supplied by the generative function. However strange it may seem, the precise period of utero-gestation in the wolf has not yet been ascertained. An erroneous statement of it was adopted by John Hunter, who employed this as an argument *against* the specific identity of the two races. But so far as is at present known the period is the same in the wolf as in the dog, namely, 63 days. If this should prove to be the case, it will be a powerful argument for their specific identity; whilst, on the other hand, if the periods should be shown to differ, the specific diversity must be admitted. The results of hybridism, too, have yet to be properly tested. It is well known that the dog will breed with the wolf, and that the offspring will breed again with either of the parent races; but it is yet to be ascertained whether the offspring of a dog and wolf will breed with another hybrid of the same kind. Should the results of this test accord with that of the preceding, no reasonable doubt, we think, will then remain on this interesting question.

Thus it will be observed that, in adopting the conclusion of the specific identity of the different races of dog with each other, the zoologist is guided by the following considerations. 1. That, notwithstanding the wide differences between the typical forms of each variety, their distinctive marks want that constancy, which is essential to a specific character. 2. That all the races breed freely with each other; the hybrid offspring breeding with one of its own kind, as readily as with one of either of the parent stocks. 3. That the period of utero-gestation is the same in all the varieties. 4. That when any of the breeds run wild for several generations, they return to a common form, losing altogether their previous distinctive peculiarities. 5. That, notwithstanding the peculiarities of habit and instinct, which the several breeds have acquired, they all possess the instinct of attachment to man; this being retained, even after the race has lived in a wild state for many generations. Upon such grounds as these, most naturalists recognise without hesitation the specifie indentity,—that is, the *common*, or at least the *similar* origin,—of all known breeds of dog; notwithstanding that the differences between them amount to well-marked changes in the form of the skull, the size of the brain, the development of the organs of sense, the form and relative size of the body, limbs, and tail, the number of vertebræ in the latter, the thickness, length, colour, and texture of the hairy covering, and peculiarities of instinct which are distinctly hereditary. And further, the approximation of the form and characters of the wild dog to those of the wolf, would be regarded, if borne out by the characters derived from the generative function, as sufficient to establish the specific identity of these two races: the wolf being the permanently wild form, the domesticated dog the humanised form, and the dingo, ghole, and other so-called *wild* races of dog, being the half-reclaimed form, of one and the same species.

For the *cause* of such variations from the common or original type, we must look in part to the original constitution of the species, and in part to the influence of external conditions. As already pointed out, there is a marked difference among various species of animals (even those nearly allied, such as the domestic cat and the tiger), in regard to their respective *capacities for variation*. It is from this circumstance, that we find some species (of plants as well as animals) restricted to particular conditions in regard to climate, food, &c., because their constitutions cannot adapt themselves to any other; whilst others are more widely dispersed, simply because their constitutions are more capable of modification in accordance with a change of circumstances. A change to which the latter can readily accomodate themselves, would be fatal to the former. Now this accommodation is effected by a change in the organism itself, of which the marks soon become evident, as we have already seen in the alteration of the hairy covering of the sheep transported to hot climates; and the continued action of the same circumstances for a few generations gives increased permanence to the new characters of the breed. It cannot be questioned, if the history of the domesticated races of animals be fairly considered, that changes in external conditions are capable of exerting a very decided influence upon the physical form, the habits and instincts, and the various functions of life, in species possessing this adaptiveness. The variations thus induced extend to considerable modifications in the external aspect, such as the colour, the texture, and the thickness of the external covering; to the structure of limbs, and proportional size of parts; to the relative development of the organs of the senses and of the psychical powers, involving changes in the form of the cranium; and to acquired propensities, which, within certain limits (depending, it would appear, on their connexion with the natural habits of the species) may become hereditary. As already remarked, wherever we find this *adaptive* capacity the greatest, we also observe the greatest tendency to what we must call *spontaneous* variation; that is, to variations of which we cannot trace any obvious cause in external conditions. Such are the varieties of features, complexion, general conformation, and mental character, amongst the offspring of the same parents, born under (so far as we can trace) the very same circumstances; and the peculiarities of family character, which are frequently thus propagated through many generations,—sometimes reappearing in the grandchildren, when they have lain dormant in the children. Such, too, are the development of additional fingers or toes, the alteration in the number of vertebræ in the tail, the unusual consolidation or separation of the toes, &c. &c.; of which we cannot find any explanation in the agency of external conditions. And we may take it as a general principle, that the tendency to *adaptive* and to *spontaneous* variation always go together.

We now come to apply the same method of investigation to the human races; and shall give a general summary of the evidence, by which, as it seems to us, the specific identity of all of them is proved as unequivocally as that of the different breeds of sheep, oxen, or pigs, whose common parentage no one at all versed in the subject ever dreams of questioning. We would remark, in the first place, that a very great diversity might naturally be looked for, amongst the races of men inhabiting different parts of the globe; since the very circumstance that individuals of any one race can sustain their existence in any part of the world where they can obtain food, shows a constitutional *adaptiveness* to external conditions, the effect of which must in the course of generations become evident in the physical conformation. And again, the variations which present themselves within the limits of any one breed or race,—that is to say, the differences which mark the several individuals, families, or nations, of whose common ancestry there is no doubt,—are greater than those which we meet with in the case of any other species. There is evidently, therefore, a very strong tendency in the human constitution to *spontaneous* variation; and it is easy to conceive that this tendency might have shown itself very early in the history of the species, so that members of the same families might have exhibited a much greater diversity than they now usually present; and, by the breeding in-and-in, which (as already remarked) would be the necessary result of a scanty and scattered population, the peculiarities thus arising would be increased and rendered permanent.

The characters which are most relied on for the discrimination of the several races of mankind, are the colour of the skin, the nature of the hair, and the conformation of the skull and other parts of the skeleton. Each of these will require to be separately considered.

The colour of the skin, it is now well known, exists in the epidermis only; and depends upon the admixture of "pigment-cells" with the ordinary epidermic cells. These pigment-cells are distinguished by their power of drawing from the blood, and elaborating in their own cavities, colouring matter of various hues; and all the varied shades of colour, presented by the different races of men, are due to the relative amount of these cells, and the particular tint of the pigment which they form. What was formerly known as the "rete mucosum," and described as a distinct colouring layer beneath the epidermis, is now known to be nothing else than the newest and softest layer of epidermis. There is no structure in the skin of the dark races that is at all peculiar to it; the very same dark cells being found in parts of the fairest skins. Still it might be affirmed, that the strongly-marked distinctions which present themselves between the typical hues of certain races, ought to be accepted as valid specific distinctions; since particular spots or patches of colour are admitted to be so in other instances. Thus, for example, the fair and ruddy Saxon, the jet-black Negro, the olive Mongolian, and the copper-coloured North American, would seem positively separated from each other by this character; propagated, as it seems to be, with little or no perceptible change from generation to generation. But although such might appear to be the clear and obvious result of comparisons of this kind, yet a more extended survey tends to break down any such distinction. For, on tracing this character through the entire family of man, we find the isolated specimens just noticed to be connected by such a series of links, and the transition from one to the other to be so very gradual, that it is impossible to say where the line should be drawn. There is nothing which at all approaches to the fixed and definite characters, which the zoologist admits as specific distinctions amongst other tribes of animals. On the other hand, we find such a constant relation between the climate and the colour of the skin, that it is impossible not to perceive the connexion between them. The parts of the globe included between the tropics or closely bordering upon them, form the exclusive seat of the native black races; whilst the colder temperate regions are the residence of the fair races; and the intermediate countries are inhabited by people of an intermediate complexion. On passing from northern Europe to central Africa, we meet with a most regular progression of this kind. Further, elevation above the level of the sea is found to have the same uniform relation to the human complexion, that it has to vegetation; for as we find the plants of temperate or even arctic regions on the sides of intertropical mountains, so do we notice that high mountains and countries of great elevation are almost uniformly inhabited by people of lighter hue than those of the surrounding country; whilst low and level countries, especially those which border on the sea, are generally tenanted by people of unusually dark colour. These distinctions are particularly well seen in Africa and in India. The deepest hue among the African races is to be found among the Negroes of the swampy plains of the Guinea coast; and there are several instances in which nations residing at no great distance from these, but at a higher level, are comparatively light, although their ancestry is undoubtedly the same. In India, there is distinct historical testimony, that races which are now found inhabiting high elevations on the Himalaya and other lofty mountain ranges, and which have complexions of almost European fairness, are the descendants of dark Hindoos, which migrated thither from the low countries.

If we take any one of those groups of nations, which are usually regarded as altogether constituting a race, such as the (so-called) Caucasian, the African, the American, or the Polynesian, we find that the greatest diversities of complexion present themselves within its limits. Thus we presume that no one who possesses even a smattering of philology will now question the eastern origin of the bulk of the European nations; yet from the very same stock that produced the fair Saxon and Celtic nations, the jet-black Hindoos of Lower India have sprung. In the same manner, upon philological evidence, the Arab tribes bordering the great desert of Sahara, some of which are as black as the darkest Negroes, are referred to the same stock with the red-haired and blue-eyed inhabitants of the mountainous regions of Yemen. Of the Jewish nation again, some which are dispersed through the colder countries of Europe, and have been acclimatized by a residence of many centuries, have come to assume the fair complexions and red or brown hair of the nations among which they live; whilst others, inhabiting Southern Europe, or still clinging to their ancient home, seem to present us with the original hue of the nation; and others, long settled in India, have become as dark as the black population around them. This last case is one of an extremely interesting character, and peculiarly satisfactory in the testimony it affords to the effect of a prolonged exposure to climatic influences; since it is well known that, in consequence of their national and religious peculiarities, the Jews maintain a complete isolation from the people among whom they may happen to dwell. It is probable that, in nations as in individuals, a pre-existing tendency to a swarthy complexion will cause the effect of long-continued exposure to a tropical climate to be more decided in blackening the hue of the skin, than it is in cases, where the fairness of the complexion shows an indisposition to the secretion of dark pigment by the epidermic cells; and that the Jews are thus more readily blackened than Saxons or Celts would be. The same holds good with regard to other nations than Jews; for the descendants of the early Portuguese settlers in India have become in many instances as dark as the Hindoos around them. No doubt, this change has originated in part from intermixture of races; but still the complete merging of the original complexion, whilst other characters of the European stock are retained, shows that such an intermixture by no means fully accounts for the change.

Various phenomena, presented by individuals of all the principal races, tend still further to break down any fancied barrier which it might be attempted to erect, on the ground of colour. For among all the chief subdivisions, *albinoism,* or the absence of pigment-cells, occasionally presents itself; so that a fair skin, nearly resembling that of the native of Northern Europe, may present itself in the offspring of the Negro or of the Red Man. On the other hand, instances are by no means rare of the unusual development of pigment-cells, in individuals of the fair-skinned races. Thus we frequently meet with persons of a swarthy complexion, whose descent appears to be quite free from the slightest infusion of the blood of the sable races, and who have never been themselves exposed to the darkening influence of hot climates. Or there may be a special development of pigment-cells in particular spots. Thus the summer freckle, the influence of sunlight in producing which cannot be questioned, is formed by an aggregation of red or brown pigment-cells. And the areola around the nipple, which in some women becomes very dark during pregnancy, derives its colour from the same source. Instances are not unfrequent, in which large patches of the surface of the body or limbs become discoloured, even almost blackened, during pregnancy; and this in females of remarkably fair complexion. The influence of light in favouring the development of the black pigment-cells is further shown by the circumstance, that the infants of dark races do not acquire their characteristic hue until some days after birth; and by the marked difference in complexion, which exists in many Polynesian nations, between the chiefs and females who pass the greater part of the day within doors or under the shelter of the umbrageous foliage, and the common people whose avocations require them to be freely exposed to the light and heat of the tropical sun; the former being often no darker than the inhabitants of Central or Southern Europe, whilst the latter are quite swarthy and sometimes even of Negro blackness. It is not improbable that such differences are partly congenital, being fixed in the respective *castes* by the operation of similar agencies through a long succession of generations; but there is no reason whatever for regarding them as indicative of original diversity of parentage. Dr. Prichard has collected a large number of cases which show that the *xanthous* variety,—distinguished by fair ruddy complexion and auburn, light brown, or red hair,—may spring up out of every melancomous or swarthy dark-haired race. In some of these cases, we find that the character of a whole tribe has changed in course of time, as may be proved by historical testimony, without the admixture of any other element, and without any obvious change in external conditions. In others, the change is confined to individuals; and this sporadic character is for the most part observable in the appearance, from time to time, of xanthous offspring from perfectly black parents. Dr. Prichard has shown that many of the so-called "white negroes" have not been *albinoes*, that is, destitute of all pigmentary matter in the skin, eyes, and hair; but have been truly *xanthous*, that is, have possessed the light colouring matter which is contained in the skin, eyes, and hair of the European. There are also cases in which fair races have become dark, without any considerable change in external conditions; thus we find that the Germanic nations, which were unanimously described by ancient authors as exceedingly fair, possessing yellow or red hair and blue or gray eyes, have become much darker since that time, so that these peculiarities are far from being common amongst them, and must now be rather looked for in Sweden. That an amelioration of the climate of Central Europe has taken place during the same period, cannot be questioned; but the climatic change scarcely seems decided enough to account for such an alteration in the physical characters of the population. In whatever way we explain the fact, however, it stands in evidence as a clear proof of the variability of particular races of men; since the purity of descent of the Germanic nations at once negatives the supposition that their change of complexion can be attributed to any admixture of a foreign element.

The characters derived from the texture and distribution of the hair are intimately connected with those furnished by its colour and by the hue of the skin; but they seem at first sight to be more permanent, and therefore more distinctive. Thus the negro is usually characterized by his "woolly" hair; whilst the Northern Asiatics, commonly referred to the Mongolian type, are equally remarkable for the scantiness and tufted arrangement of their pilous covering, which has been compared to the mode in which the bristles are set in a scrubbing-brush. But, however decided these characters may seem to be in extreme cases, their value as indications of original difference of race altogether disappears when we examine closely into them. For, in the first place, Dr. Prichard has clearly shown, by microscopic examination, that the hair of the Negro is not really wool, and that it differs in regard to its intimate structure from that of the fairer races, solely in the greater quantity of pigmentary matter which it contains in its interior; and, in this respect, it is similar to the jet-black hair not unfrequently to be met with in our own country. The crisp and twisted growth of the Negro hair is the only character by which it can be really separated from the straight, flowing hair common amongst European nations; and this alone cannot be relied upon for a moment as an indication of original difference, since these national variations do not exceed those which present themselves within the limits of any one race. Thus we occasionally meet with Europeans whose hair is nearly as "woolly" as that of most Negroes; whilst, on the other hand, if we take the entire mass of the black native races of Africa into comparison, we shall find tribes among them, which, though similar in complexion and in most other physical peculiarities, yet differ in regard to their hair, and present every possible gradation from a completely crisp or so-called "woolly" hair, to merely curled and even to flowing hair. A similar statement may be made respecting the inhabitants of the islands of the great Southern Ocean; some individuals having crisp hair, whilst others of the same race have it merely curled. Even if, as Dr. Prichard justly remarks, the hair of the Negro were really analogous to wool, it would by no means prove the Negro to be a peculiar and separate stock, unless the peculiarity were *constantly* presented by all the nations of Negro descent, and were restricted to them alone; for there are breeds of domesticated animals which bear wool, whilst others of the same species, under different climatic influences, are covered with hair.

By most writers on the diversities of mankind, the varieties which are observable in the form and structure of the bony skeleton, and particularly in the cranium and pelvis, have been thought to furnish more important characters for the separation of the races, than those derived from the colour of the skin or the texture of the hair,—a certain capability of alteration in which, under climatic influence, can scarcely be gainsaid. It might certainly be supposed *à priori* that strongly-marked peculiarities in the configuration of the skull, in the proportion of the parts of the body, and in the development of the brain, would be less likely to undergo modification from changes in external conditions or from spontaneous variation, than are the outward or superficial marks furnished by the tegumentary covering and its appendages. Since the time of Camper and Blumenbach, various attempts have been made by anatomists to divide mankind into groups, by taking the shape of the skull as the chief ground of distinction; but scarcely any two writers have agreed as to the number of primary divisions that should be established; and in nearly all such attempts it has been assumed as a guiding principle, that conformity in physical characters must be taken as the best indication of family relationship amongst nations,—a position which later philological investigations seem to have overthrown. It would be easy to select a European, an African, a Mongolian, a Malay, and an American skull, which, when strongly contrasted, might afford sufficiently distinctive characters; but the question would then arise, whether these characters are common to the entire races which they have been selected to represent, and whether they have the degree of permanency and invariability which is requisite to enable us to regard them as a ground of specific distinction. Now it will appear from the investigations, of which we shall subsequently give an outline, that no such typical forms can really be invested with any such character of constancy; for that they are far from being invariably presented in the several nations of one race, or even in the several individuals of one nation; and that they, too, are liable to undergo alteration under the influence of change in external conditions. It does not appear, however, that such alterations are liable to take place under the mere agency of climate; but the general habits of life, the supply of food, and the mode and degree of exercise of the mental faculties,—every influence, in fact, which enters into the terms barbarism and civilization,—have a much greater tendency to bring them about. And consequently we find that in every race there may be an approximation, both in the form of the skull and in the general configuration of the body, to the characters which we are accustomed to regard as distinctive of the highest; whilst the highest may undergo such degradation as may bring it into approximation with some of those usually ranked as inferiors. The number of leading types of configuration of the skull is reduced by Dr. Prichard to *three;* and he considers that there is sufficient evidence for connecting these with different habits of life.

"Amongst the rudest tribes of men, hunters and savage inhabitants of forests, dependent for their supply of food on the accidental produce of the soil or on the chase, among whom are the most degraded of the African nations and the Australian savages, a form of head is prevalent which is most aptly distinguished by the term *prognathous*, indicating a prolongation or extension forward of the jaws; and with this characteristic other traits are connected which will be described in the succeeding pages.

"A second shape of the head, very different from the last mentioned, belongs principally to the nomadic races, who wander with their herds and flocks over vast plains, and to the tribes which creep along the shores of the Icy Sea, and live partly by fishing and in part on the flesh of their reindeer. These nations have broad and lozenge-formed faces, and what I have termed *pyramidal* skulls. The Esquimaux, the Laplanders, Samoides, and Kamtschatkans, belong to this department, as well as the Tartar nations, meaning the Mongolians, Tungusians, and nomadic races of Turks. In South Africa, the Hottentots, formerly a nomadic people who wandered about with herds of cattle over the extensive plains of Kafirland, resembling in their manner of life the Tungusians and the Mongoles, have also broad-faced, pyramidal skulls, and in many particulars of their organization resemble the Northern Asiatics. Other tribes in South Africa approximate to the same character, as do many of the native races of the New World.

"The most civilized races, those who live by agriculture and the arts of cultivated life, all the most intellectually improved nations of Europe and Asia, have a shape of the head which differs from both the forms above mentioned. The characteristic form of the skull among these nations may be termed *oval* or *elliptical*." (Natural History of Man, p. 108.)

Now that these typical forms are *not* permanent, but may be changed under the influences of civilization,—just as we have seen the form of the cranium to be changed by domestication in the hog, to say nothing of the greater changes in the canine races,—appears to us to be demonstrated by adequate evidence. Although it is not infrequent to cite the Negro race as an example of *permanence* under different conditions, yet nothing can be more fallacious. We have the testimony of a most intelligent observer, Dr. Hancock of Guiana, to the fact that it is not uncommon to meet with Negroes in that colony, the descendants of those first introduced into it, who now exhibit a decidedly European cast of countenance, although their descent has been perfectly free from admixture of European blood; and he even goes further and asserts that, among those especially who have been employed as house-servants, and have therefore been brought into closer relation with their European masters, it is not at all difficult to distinguish a Negro belonging to the Dutch portion of the colony from another belonging to the British settlements, by the correspondence in the features and expression of each with those which are characteristic of his respective masters. We have been informed by Mr. Lyell that, during his recent tours in the United States, he made numerous inquiries on this head, especially from medical men residing in the slave states; and that the testimony of all those who had paid attention to the subject was unanimously to the same effect,—the approximation in general configuration of head and body to the European model being more and more evident in each successive generation of Negroes, especially in such as were brought into closest and most habitual relation with the whites, without any actual intermixture of races. There is no nation in which we have the power of tracing historically the elevation of the skull from the prognathous type to the perfect oval or elliptical, under the influence of civilization prolonged through many successive generations; but as the most elevated physical characters are found amongst those of the African nations, which have, in some degree, emerged from their original barbarism, chiefly in consequence of the superior influence of the Mahommedan religion which they have embraced, it does not seem unfair to presume that their elevation has been the result of their refinement. On the other hand, the most strongly-marked examples of the prognathous type are to be found amongst the most degraded Negroes of the Guinea Coast, and among the Australians, who may be ranked with these as very near the bottom of the scale of humanity.

The variability of the *pyramidal* type of skull, and its convertibility into the more symmetrical type, characteristic of the so-called Caucasian race, seems clearly demonstrated by the change which a portion of the Turkish nation has undergone. The present aspect of the cranium and features of the

Turks of Europe and Western Asia is, on the one hand, so closely accordant with that which we encounter in the great bulk of European nations, and on the other, so dissimilar to that which is presented by the Turks of Central Asia, that some of those writers, who look upon physical characters as the chief test of family relationship amongst nations, have actually maintained the Western or Ottoman Turks to be descendants of the Caucasian stock, and to have no real affinity with the Eastern or Mongolian race. All the most learned inquirers, however, are now agreed that there is clear historical evidence that the Ottoman Turks are a branch of the same stem with the Turks of Central Asia. The latter continue in the original nomadic habits of the nation; and continue to exhibit, probably with little or no alteration, the original configuration of the skull and characteristic physiognomy of the race. The former, however, having conquered the countries which they now inhabit eight centuries since, have settled down to the fixed and regular habits of the Indo-European nations, and have made corresponding advances in civilization; and the national type of cranium has been completely changed during this period, from the *pyramidal* to the *elliptical.* Some of those who have been forced to admit the fact of this change, have endeavoured to avoid what seems the obvious inference from it,—that the change has been the result of civilization and social improvement,—by attributing it to an intermixture of the Turkish race with that of the countries which they conquered, or to the introduction of Georgian or Circassian slaves into their harems. But there is every probability that the difference of religion and manners kept them as separate at first, as it has since done, from the inhabitants of the countries which they conquered in the course of their migration; and the improvement effected by the introduction of Georgian and Circassian slaves must have been confined to the higher classes who alone can afford to purchase them. In either case, the cause assigned, even if admitted to the full extent claimed, would have been insufficient to produce the entire substitution of the new type for the original one, which observation demonstrates to have taken place. There is one other attempt at explanation which requires notice, although it has not the least pretence to be so received. There are those who maintain that the entire Turkish race is of Caucasian origin; the type of conformation now presented by the Ottoman Turks being the original; whilst the tribes of Central and Eastern Asia, which now present the pyramidal skull, have acquired that type by admixture with the Mongolian races. Here, again, the explanation would be insufficient, even if the facts were most fully admitted, to account for the *complete* substitution in the Tartar races of the pyramidal type for the elliptical form, which, according to this hypothesis, they originally possessed. But the idea is totally destitute of foundation. All historical testimony goes to prove that the race primarily sprang from the remote East, and that it was entirely disconnected from the Indo-European races until the westward migration of a portion of it brought it into collision with them. It is utterly absurd to imagine that its original character should have been preserved better by those parts of the race which have undergone a complete change in their habits of life, than amongst those which continue in their primitive semi-barbarous condition. And the supposition that any extensive change could have been produced by an admixture of the Mongolian element, is at once negatived by the fact that the Mongols form but a small tribe in comparison with the widely-extended nations of the Turkish race; whilst the purity of descent of the latter is further evidenced by the small amount of variation in the languages even of those most removed from each other,—their conformity being so close that, according to M. Jaubert, a native of Constantinople might travel eastwards as far as the frontier of the Chinese empire, and be understood all the way.

Another instance of the same modification is to be found in a different group of the nations originally characterized by pyramidal skulls. The North and East of Europe still contains the descendants of tribes, which seem to have been in possession of it before the appearance of the races of Indian descent in that quarter of the globe. Some of these are well known under the name of Lapps and Finns, whose similarity of origin is indubitable, although their physical characters now present important differences; the Lapps retaining, in a well-marked degree, the pyramidal skull, whilst the form of the cranium, in the modern Finn, is far more oval. But there is another group not commonly referred to the same stock, which appears from Dr. Prichard's researches to be fairly referable to it, and to have undergone a still greater departure from the original type; this is the race of Magyars, of which the Hungarian nobility is composed, a race which is not inferior in physical or in mental characters to any in Europe. Now we have in the Lapps, Finns, and Magyars, three distinct gradations in the form of the cranium, between the pyramidal and the elliptical type; and the amount of change, in each tribe, presents a most striking conformity to its degree of advance in civilization. Thus the Lapps, retaining the original nomadic habits of the race, retain also the pyramidal type more decidedly than either of the others; whilst the Finns, who are nothing else than Lapps half civilized by a settled agriculture, show a decided approximation to the ordinary European physiognomy; and in the Magyars, who have made the greatest advances in refinement, that which may fairly be presumed to have been the original type has been entirely superseded by the more elevated one.

On the other hand, that the continued operation of degrading causes tends to produce a degeneration in the form of the cranium, and in the physiognomy of the more elevated races, as well as in their general configuration, must be apparent, we think, to those who have had opportunities of studying the physical characters of the worst specimens of our own population. Dr. Prichard quotes from an Irish writer a very striking testimony to the truth of this position; and we quote it in full, as being of peculiar importance at the present time, when public attention is being so strongly directed to the social evils of that unhappy country, which threaten, if continued, to reduce the great mass of the population to the same fearful state as that here so graphically portrayed.

"On the plantation of Ulster, and afterwards on the successes of the British against the rebels of 1641 and 1689, great multitudes of the native Irish were driven from Armagh and the south of Down, into the mountainous tract extending from the barony of Flews eastward to the sea;—on the other side of the kingdom, the same race were expelled into Leitrim, Sligo, and Mayo. Here they have been almost ever since, exposed to the worst effects of hunger and ignorance, the two great brutalizers of the human race. The descendants of these exiles are still readily distinguishable from their kindred in Meath and in other districts where they are not in a state of physical degradation; being remarkable for *open projecting mouths, with prominent teeth and exposed gums; their advancing cheek-bones and depressed noses bearing barbarism on their very front.* In Sligo and northern Mayo, the consequences of two centuries of degradation and hardship exhibit themselves in the whole physical condition of the people; affecting not only the features but the frame, and giving such an example of human deterioration from known causes, as almost compensates, by its value to future ages, for the suffering and debasement which past generations have endured in perfecting its appalling lesson. Five feet two inches upon an average, pot-bellied, bow-legged, abortively-featured, their clothing a wisp of rags,—these spectres of a people, that were once well-grown, able-bodied, and comely, stalk abroad into the daylight of civilization, the annual apparitions of Irish ugliness and Irish want. In other parts of the island, where the population has never undergone the influence of the same causes of physical degradation, it is well known that the same race furnishes the most perfect specimens of human beauty and vigour, both mental and bodily." (Dublin University Magazine, No. 48.)

The characters here assigned to a certain section of the Irish people are so precisely those which, in degrees more or less exaggerated, are presented by the natives of Australia, usually considered to be amongst the lowest in the scale of humanity; that it is not possible to avoid the inference that the latter owe their physical inferiority, in great part, to the extreme degradation of their mode of existence. We were particularly struck with the resemblance, on happening to see not long since the representation of a group of the natives of Australia, which is among the plates illustrating the Voyage de l'Astrolabe. This group would have passed very well, in all but their clothing and decorations, for an assemblage of such Irish as have been just described, specimens of which not unfrequently present themselves in our large towns.

There is another race at present existing under circumstances of the most depressing kind, and exhibiting the utmost degradation in its physical characters; in which the progress of the descent from a much higher physical and social condition can be historically traced. Writers on the history of mankind seem to be nearly agreed in considering the Bushmen or Bosjesmen, of South Africa, as the most degraded and miserable of all nations, excepting, perhaps, the Australian savages. They are described by M. Bory de St. Vincent, as differing most widely from what he terms the Japetic species of men, and as forming the transition from the genus Homo to the genera of Orangs and Gibbons; he even finds analogies between them and the Macacos. He speaks of them as even too brutish to be reduced to slavery; as having a language consisting only of a few guttural tones, and incapable of expressing any but a very limited range of ideas; and as scarcely having a capacity for reasoning. Their present mode of life is thus described by Dr. Prichard:

"No picture of human degradation and wretchedness can be drawn, which exceeds the real abasement and misery of the Bushmen, as we find it displayed by the most accurate writers who describe this people. Without houses, or even huts, living in caves and holes in the earth, these naked and half-starved savages wander through forests, in small companies or separate families, hardly supporting their comfortless existence by collecting wild roots, by a toilsome search for the eggs of ants, and by devouring, whenever they can catch them, lizards, snakes, and the most loathsome insects." (Natural History of Man, p. 515.)

There is nothing, however, in the form of their skulls or in their physiognomy, which departs more widely from that of the Hottentots—to whom, as we shall presently see, they are nearly related,—than the physical characters of the most degraded tribes of the Irish nation differ from those of the superior part of the same nation; and there is not, as we are assured by unprejudiced observers, anything like that deficiency in their moral and intellectual faculties, which has been represented to exist by those who are searching for approximations between mankind and the inferior species of animals. Mr. Burchell, who sought and obtained opportunities of conversing with them, and of observing their manner of existence, though he found them in the most destitute and miserable state, yet discovered among them traits of kind and social feeling, and all the essential attributes of humanity. But whatever may be their *present* amount of degradation, the general argument in favour of the influence of external conditions is rather strengthened than weakened by those who depict it in its strongest colours; since there is clear proof that the Bushmen do not constitute a distinct race, but are nothing else than degenerated Hottentots.

"A careful comparison of their language with that of the Korah and other Hottentots, convinced Professor Vater that there is an essential affinity between them; and in recent times this conclusion has been fully established by local inquiries, and no diversity of opinion at present exists upon the subject. We are assured by one of the latest and best writers on South Africa, that the Bushmen are the remains of Hottentot hordes, who subsisted originally, like all the tribes of Southern Africa, chiefly by rearing sheep and cattle; but who have been driven by the gradual encroachments of European colonists, and by internal wars with other tribes, to seek for refuge among the inaccessible deserts and rocks of the interior. 'Most of the hordes,' says the same writer, 'known by the name of Bushmen, are entirely destitute of flocks and herds, and subsist partly by hunting, partly on the wild roots of the wilderness, on reptiles, locusts, and the larvæ of ants, or by plundering their hereditary oppressors, the colonists of the frontier. Having descended from the pastoral to the state of robbers and hunters, the Bushmen, as we are assured, have necessarily acquired, with their increased perils and privations, a more resolute and ferocious character: from a mild, confiding, and unenterprising race of shepherds, they have been gradually transformed into wandering hordes of fierce, suspicious, and vindictive savages; by their fellow-men they have been treated as wild beasts, until they have become assimilated to wild beasts in their habits and dispositions.'

"Difficult as it may be to imagine a change from the state of herdsmen to that of the miserable Bushmen, the transition has been actually observed and described. Among the Hottentot tribes, the Koranas are well known to be the most advanced in all the possessions and improvements which belong to the pastoral life. A late traveller in Africa, whose narrative is replete with good sense and the marks of accurate knowledge, has traced from observation the process by which hordes even of the Korah race have been reduced from the life of peaceful herdsmen to the condition of hunters and predatory savages. The Koranas, as visited by Mr. Thomson on the Hartebeest river, had actually undergone this transition; having been plundered by their neighbours and driven out into the wilderness to subsist on wild fruits, they adopted the habits of the Bushmen, and had become assimilated in every essential particular to that miserable tribe." (Natural History of Man, p. 517.)

We cannot suppose that any properly civilized race could undergo such a degeneration, in the course of the one or two generations which seem sufficient to convert Hottentots into Bushmen. But it is to be borne in mind, that the first steps in the progress by which a nation emerges from barbarism are not towards those attainments in literature and art which, when once acquired, can never entirely lose their influence ; but towards improvements in the simplest arts of life, the cultivation of the ground and the rearing of cattle, the construction of habitations, and the manufacture of implements. If deprived of these, they are at once depressed into a condition worse perhaps than their primitive rudeness; for they have not yet attained the intellectual development which will enable them to rise superior to circumstances and to triumph over difficulties apparently the most insuperable. That the moral and intellectual nature of the Hottentots is not different from our own, and though at present inferior is capable of unlimited elevation, will sufficiently appear, we think, from considerations hereafter to be stated; and every shadow of a reason, therefore, for regarding the Bushmen as one of those distinct races which are separated from our own by an impassable barrier, disappears under the light of a careful scrutiny of the facts. And if this be the case with regard to the wretched Bushmen, how much less ground is there for entertaining such a notion regarding the Negroes; whole nations amongst whom have attained a considerable degree of civilization; whilst individuals have every now and then proved by their attainments in the literature, art, and science of Europe, that they possess every faculty boasted by the so-called superior races, and that too in no inferior degree. The comparative rarity of such individuals must be imputed to two causes : first the want of opportunity for improvement, to which the great bulk of the race is at present condemned; and secondly, an inherent inferiority. This inferiority may be freely admitted by the warmest advocates of the equal rights of nations; for the admission involves no contradiction or abatement of their principles. That the Bushman and the Negro are at present on the average inferior in intellectual and moral capacity to the average of Englishmen, Frenchmen, or Yankees, only shows, in our apprehension, that they have been longer subjected to the influence of degrading causes; whilst the elevation occasionally attained by individuals clearly demonstrates that the capacities of these races have not been permanently and hopelessly repressed, but are capable of being again developed.

We have digressed in some degree from our direct line of inquiry ; but differences in the form of the cranium have such an intimate connexion with the general capabilities of the several races which present them, and have been so much dwelt upon as establishing valid specific distinctions, that we have thought it necessary to enter at some length into the proof that these differences may be produced, or at least greatly modified, by the influence of external agencies prolonged through a sufficient period. We shall now advert to certain differences in the form of the pelvis, which have been thought to be characteristic of particular races or groups of nations. According to Dr. Vrolik of Amsterdam, the difference between the male and female pelvis is much more strongly marked in the Negro than in the European. The pelvis of the male Negro resembles, in the strength and density of its substance and of its component bones, the pelvis of a wild beast; but the pelvis of the female in the same race combines lightness of substance and delicacy of form and structure, whilst it presents a conformation which approximates in his estimation towards the narrow elongated pelvis of the chimpanzee or orang. The structure of the pelvis in the Hottentot race is only known from the skeleton of the female who died in Paris in 1815; the shape of which indicated, in Dr. Vrolik's opinion, a still greater animality than is found in the pelvis of the Negro. In no other individual, except from deformity, have the ilia been observed to assume so vertical a direction; and they are remarkable, likewise, for their great height in proportion to their breadth, which helps to give the pelvis its character of peculiar elongation. Dr. Vrolik further states that the pelvis of the Javanese is distinguished by its peculiar lightness of substance, the smallness of its size, and the circular form of its upper opening. A comparison of the same portion of the skeleton in different nations has been carried out, however, upon a more extended and philosophical plan by Professor Weber of Bonn. Bringing together all the pelves he can collect, he classifies them according to their shape; and finds that they fall into four principal divisions, as follows:

"1. The oval form—*die ovale ur-becken-form.* An oval pelvis is one in which the upper opening presents an egg-shaped figure in such wise that this aperture at the anterior part, namely, at the symphysis pubis, is narrow; but towards the middle of the same aperture and the junction of the ilia with the os sacrum, becomes gradually and proportionally widened, and again becomes somewhat narrower in passing backwards towards the promontorium, when it ends in an obtuse point.

"2. The round form of the pelvis. A round pelvis is one in which the upper opening is round. The circumference, particularly at the symphysis and horizontal branches of the pubis, is more spread out than in the round oval form, whereas the conjugate has nearly the same extent as the transverse diameter.

"3. The square or four-sided form is the shape of a pelvis of which the sides, especially that formed by the os pubis, are flat and broad, so that the upper opening forms nearly a perfect square: the transverse diameter is greater than the conjugate.

"4. The wedge-shape—*keil-formige ur-becken-form*—belongs to the pelvis which appears on both sides compressed, so as to be narrower from side to side than from front to back. The ossa pubis unite under an acute angle, and the horizontal branches run backwards in a straighter direction than in the oval form; the conjugate is lengthened, and the upper opening is oblong rather than oval." (Natural History of Man, p. 127.)

Now if an attempt be made to assign one of these forms of pelvis to any particular nation or group of nations, as a constant distinctive character, it is found to fail *in toto;* specimens of each kind being found in the same races, although particular types are more common than others in particular races. Thus the oval type is more common in the European, where it is in accordance with the oval form of the skull; but round, square, and wedge-shaped pelves also occur amongst individuals of that race; whilst the oval form presents itself in the pelvis of a Botacudo, a people reputed to be the most savage of all the American nations. The round type is most frequent among the American nations; but it has been observed by Weber not merely in the European, but in a Negress, a female Hottentot, and a Javanese. The square form seems most common among the nations with pyramidal skulls, commonly ranked as Mongolian; but it occurs also in Europeans and in the mixed race of Mestizos. The oblong or wedge-shaped pelvis is most common amongst the African races, and is conformable to the elongated shape usually possessed by their crania; but it has been observed also among Europeans and Botocudos.—Thus even from the very imperfect materials yet collected, it may be deduced with certainty that the form of the pelvis affords no character of sufficient constancy to establish well-marked distinctions amongst the races of mankind.

Other characters which have been at different times adduced, as showing that the Negro and other degraded races are really to be considered as forming a distinct group, intermediate between the higher specimens of humanity and the superior apes, are found on close examination to be equally insufficient. Well-marked differences undoubtedly present themselves when we compare the average of one race with the average of another; but *individuals* are found in every tribe, who in all the particulars in question *pass* the intermediate line, and agree with the tribe which is distinguished from the majority of their own kindred. Thus, in the Negro, the bones of the leg are much more convex in front than in Europeans, so as to give the limb that peculiar shape which is commonly known as the cucumber-shin; and the foot is much less arched, being broad and flat. But the same conformation not unfrequently presents itself in individual Europeans; whilst individuals are occasionally found among the Negroes, whose persons might be taken as models of symmetry and vigour; witness the celebrated athlete, a cast of whose body is conspicuously displayed in the Museum of the College of Surgeons. Again, it was stated by White, and has been generally believed, that the length of the fore-arm in the Negro is so much greater than in the European, as to constitute a real approximation to the character of the Apes. But an extended comparison proves that only a very slight difference exists between the *average* of each race; and that this difference is by no means greater than those which may be observed on comparing the individuals, of which any single race or nation is composed. On the other hand, a constant and decided difference exists in this particular between Man and the highest Apes; for whilst the hands of man do not hang, when he is erect, below the middle of the thighs, (except in cases of absolute deformity, as from curvature of the spine), those of the chimpanzee extend below the knee-joint, and those of the orang as far as the ankle. So, again, it has been a general opinion since the time of Soemmering, that the head of the Negro is placed so much further back on the vertebral column, as to occasion a material difference in the figure of the whole body, causing an approximation to the posture of the lower animals, in which the foramen magnum is placed considerably behind the centre of gravity. The posterior situation of the foramen magnum, however, in the Negro skull, as compared with that of the European, is more apparent than real; being chiefly dependent upon the projection of the upper jaw, which adds to the portion of the cranium in front of the aperture, whilst its position in reference to the cranial cavity has undergone no change. And we meet with a most decided alteration in the site of this aperture, when we examine it in the adult orang or chimpanzee; although in the young of these Apes it is not so apparent.

We have now brought under examination every one of the characters which have been relied upon by those naturalists, who would divide mankind into a certain number of races, having had distinct origins, and continuing to be separated by well-marked physical characters. We have shown that neither the colour of the skin, the colour or texture of the hair, the shape of the skull, the conformation of the features, or the general configuration of the body, whether considered singly or in combination, afford characters of sufficient fixity to be employed, according to the canons of Natural History, as valid specific distinctions. We have seen that they are all liable to be affected by the influence of external conditions, especially if these influences are prolonged through successive generations; and that whatever is not capable of being accounted for in this manner, may be legitimately set down to that tendency to spontaneous variation in the human race, of which almost any family of half a dozen children will afford abundant illustration. We consider that the investigation, of which we have thus given a sketch, leaves those upholders of the diverse origin of the human races, who contend for the fixity of the characters at present possessed by them, without the least foundation for their argument; and affords strong *negative* testimony in favour of the community, or at least of the similarity of their parentage. For the *positive* argument on the same side, together with a brief account of the principal groups into which the human family is distributed by Dr. Prichard, we must refer our readers to our next Number; in which, too, we shall say something of the distinctive characters of the two works at the head of our article, and shall endeavour to express our sense of the vast obligations under which we conceive that both science and philanthropy have been laid by the persevering devotion manifested by Dr. Prichard through his entire professional life, to this one great object; than which nothing can well be conceived to be less remunerative, either directly or indirectly, when weighed in that commercial balance by which we are too much accustomed to estimate the merit of our pursuits.

---

